# Presence of Hydrogen Peroxide, a Source of Hydroxyl Radicals, in Acid Electrolyzed Water

**DOI:** 10.1371/journal.pone.0046392

**Published:** 2012-09-28

**Authors:** Takayuki Mokudai, Keisuke Nakamura, Taro Kanno, Yoshimi Niwano

**Affiliations:** Tohoku University Graduate School of Dentistry, Sendai, Japan; University of Wisconsin-Milwaukee, United States of America

## Abstract

**Background:**

Acid electrolyzed water (AEW), which is produced through the electrolysis of dilute sodium chloride (NaCl) or potassium chloride solution, is used as a disinfectant in various fields because of its potent antimicrobial activity. The hydroxyl radical, an oxygen radical species, is often suggested as a putative active ingredient for AEW antimicrobial activity.

**Methodology/Principal Findings:**

The aim of the present study is to detect hydroxyl radicals in AEW. The hydroxyl radicals in AEW prepared under different conditions were determined using an electron spin resonance (ESR) technique. A signal from 5,5-dimethyl-1-pyrroline *N*-oxide (DMPO)-OH, an adduct of DMPO and the hydroxyl radical, was detected in AEW prepared by double or triple electrolyses of 1% NaCl but not of 0.1% NaCl solution. Then the presence of hydrogen peroxide as a proposed source of hydroxyl radicals was examined using a combination of ESR and a Fenton reaction. The DMPO-OH signal was clearly detected, even in AEW prepared by single electrolysis of 0.1% NaCl solution, when ferrous sulfate was added to induce a Fenton reaction, indicating the presence of hydrogen peroxide in the AEW. Since sodium formate, a hydroxyl radical scavenger, did not affect the bactericidal activity of AEW, it is concluded that the radical is unlikely to contribute to the antimicrobial activity of AEW, although a small amount of the radical is produced from hydrogen peroxide. Dimethyl sulfoxide, the other hydroxyl radical scavenger used in the present study, canceled the bactericidal activity of AEW, accompanied by complete depletion of free available chlorine, suggesting that hypochlorous acid is probably a major contributor to the antimicrobial activity.

**Conclusions:**

It is strongly suggested that although hydrogen peroxide is present in AEW as a source of hydroxyl radicals, the antimicrobial activity of AEW does not depend on these radicals.

## Introduction

Acid electrolyzed water (AEW), which is produced by electrolysis of dilute sodium chloride (NaCl) or potassium chloride solution in the anode side of an instrument where the anode and cathode are separated by an ion-permeable diaphragm, has been used mainly in the agricultural and medical fields as a disinfectant in farm and food hygiene [1]–[8], and disinfection of medical instruments such as dialyzers [9], patient-used endoscopes [10], and dentures [11] because of the potent antimicrobial potential of AEW [12]–[16]. The antimicrobial activity of AEW might be the result of the combined effects of the oxidation–reduction potential (ORP) and pH [17] because the characteristic values of AEW such as low pH (2.7 or lower) and high ORP (+1100 mV or higher) deviate from the tolerable range for microbial growth (pH 3–10, ORP +900−400 mV) proposed by Becking et al. [18]. In addition, the hydroxyl radical, an oxygen radical species, is often suggested as a putative active ingredient for AEW antimicrobial activity [19]. To date, the main contributors to the antimicrobial activity of AEW have been thought to be hypochlorous acid (HClO) with a low pH, and a high ORP [20]. Some studies have also suggested that HClO, as an undissociated form of chlorine, penetrates microbial cell membranes and subsequently achieves its antimicrobial action through oxidation of key metabolic enzymes [21]–[23].

No clear evidence has been obtained for oxygen free radicals as putative contributors to the antimicrobial activity of AEW. In a study where an ESR spin-trapping technique was applied to AEW using two spin traps, 5,5-dimethyl-1-pyrroline N-oxide (DMPO) and N-[(1-oxido-4-pyridinio)methylene]-t-butylamine N-oxide, the spin adducts of hydroxyl radical were observed when electrolyte was dissolved in tap water but not in pure water [24]. By contrast, in a similar study where the ESR spin-trapping technique was used to analyze AEW, no detectable spin adducts of oxygen free radicals were observed [25]. More in detail, the addition of DMPO to AEW generated an ESR signal from 5,5-dimethyl-2-pyrrolidone-N-oxyl (DMPOX) but not ESR signals from DMPO-OH (a spin adduct of DMPO and a hydroxyl radical) and DMPO-OOH (a spin adduct of DMPO and a superoxide anion), suggesting that hypochlorous acid oxidizes the spin-trap DMPO, with the formation of DMPOX. In a recent review article, it was also proposed that bactericidal activity of AEW is mainly dependent on available chlorine in the form of HClO and molecular chlorine (Cl_2_), but not radicals [26]. In the present study, we hypothesized that a proton, molecular oxygen, and an electron generated through electrolysis of the electrolyte solution in the anode side can result in the formation of hydrogen peroxide (O_2_+2H^+^+2e^−^→H_2_O_2_) and subsequently hydroxyl radicals. To confirm the presence of hydrogen peroxide and the hydroxyl radical, we used a combination of an ESR spin-trapping technique and a Fenton reaction, which can be used as a tool for determining hydrogen peroxide [27].

## Results

### Characteristic values and ESR analysis of AEW

**Figure 1:**
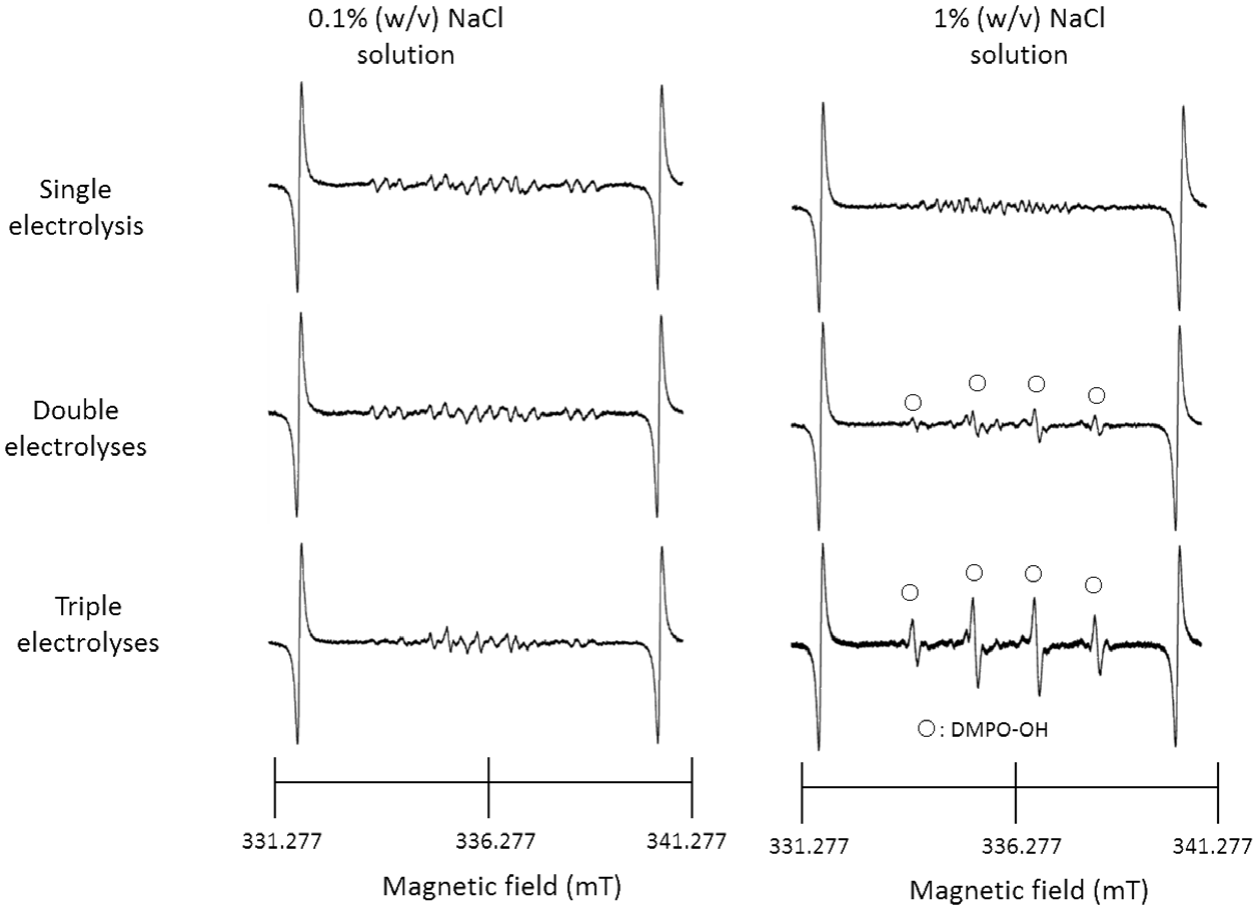
ESR spectra of AEW from single and repeated electrolyses of 0.1% and 1% (w/v) NaCl solutions.

**Figure 2:**
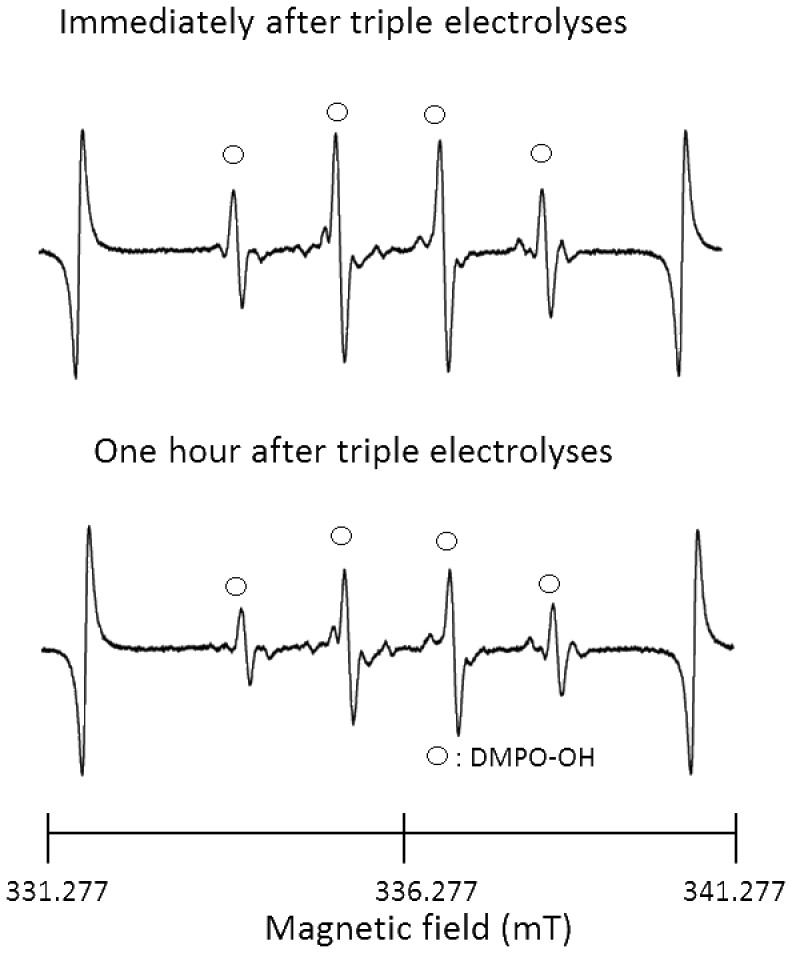
ESR spectra of AEW from triple electrolyses of 1% (w/v) NaCl solution. Spectra immediately after production and after 1-h storage at room temperature under light-shielding conditions are shown.

The characteristic values of AEW obtained by single and repeated electrolyses of 0.1% (w/v) NaCl solution and 1% (w/v) NaCl solution are summarized in Tables 1 and 2, respectively. In the case of single electrolysis of 0.1% (w/v) NaCl solution, the pH, ORP, and residual chlorine concentration were 2.43, 1179 mV, and 66 mg/L, respectively. When AEW from 0.1% (w/v) NaCl solution was subjected to up to two more electrolyses, the pH tended to decrease, and both the ORP and the residual chlorine concentration tended to increase. Similar tendencies were observed in AEW from 1% (w/v) NaCl solution, with apparently higher concentrations of residual chlorine than those from 0.1% (w/v) NaCl solution. In a separate experiment where dissolved oxygen was determined in AEW, irrespective of the NaCl concentrations as the electrolyte, dissolved oxygen concentrations increased with the number of electrolysis from approximately 0.25 to 0.35 mM under the identical conditions to those in Tables 1 and 2. Representative ESR spectra of the AEWs described in Tables 1 and 2 are shown in Fig. 1. In the AEW from 0.1% (w/v) NaCl solution obtained by single and double electrolyses, no clear signal from DMPO-OH was detected. However, a DMPO-OH-like signal, possibly with a DMPOX signal, appeared after three electrolyses. When 1% (w/v) NaCl solution was used as the electrolyte solution, a clear signal from DMPO-OH was detected in the AEW, especially that obtained by triple electrolyses, and the concentration of DMPO-OH was calculated to be 6.2 µM, using 20 µM 4-hydroxy-2,2,6,6-tetramethylpiperidine (TEMPOL) as a standard. The spin adduct DMPO-OH was assigned using hyperfine coupling constants (hfcc). The hfccs are *a*H = *a*N = 1.49 mT, which coincide with those of the DMPO-OH adduct reported in a previous paper [28]. Since the half-life of a hydroxyl radical is extremely short [29]–[31], there is a possibility that the radical disappears rapidly. Thus, to determine whether the hydroxyl radical remains in the AEW after electrolysis, AEW obtained by triple electrolyses was stored for 1 h at room temperature under light-shielding conditions. Representative ESR spectra of the AEW immediately and 1 h after the third electrolysis are shown in Fig. 2. The DMPO-OH signal was detected even after storage for 1 h.

**Table 1:** Characteristic values of AEW obtained from 0.1% NaCl solution.

The number of electrolysis	pH	ORP (mV)	Residual chlorine* concentration (mg/l)
1	2.43	1179	66
2	2.29	1194	124
3	2.14	1202	180

Each value represents the mean of duplicate determinations.

* Residual chlorine is consisted of combined available chlorine and free available chlorine.

**Table 2:** Characteristic values of AEW obtained from 1% NaCl solution.

The number of electrolysis	pH	ORP (mV)	Residual chlorine* concentration (mg/l)
1	2.51	1167	230
2	2.31	1179	390
3	2.16	1184	500

Each value represents the mean of duplicate determinations.

* Residual chlorine is consisted of combined available chlorine and free available chlorine.

### Induction of Fenton reaction to determine hydrogen peroxide

**Figure 3:**
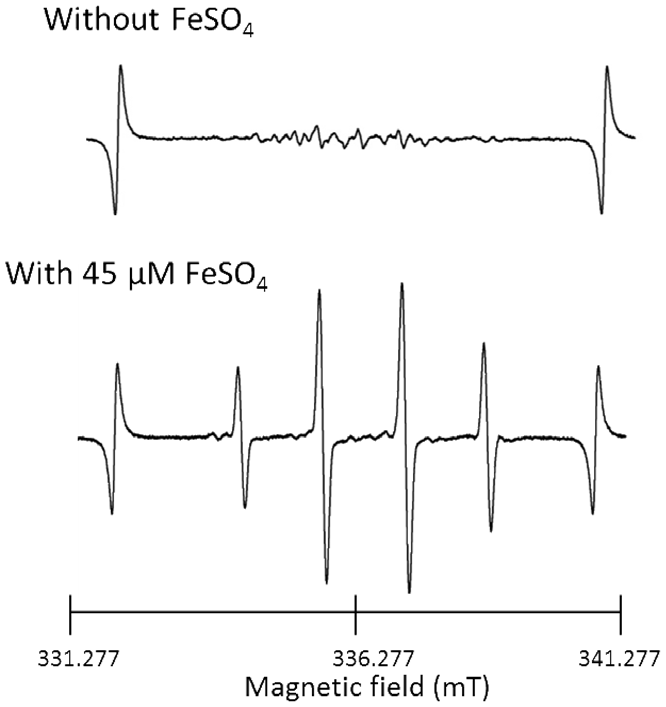
ESR spectra of AEW prepared by single electrolysis of 1% NaCl solution. Spectra in the absence and presence of FeSO_4_ are shown.

**Figure 4:**
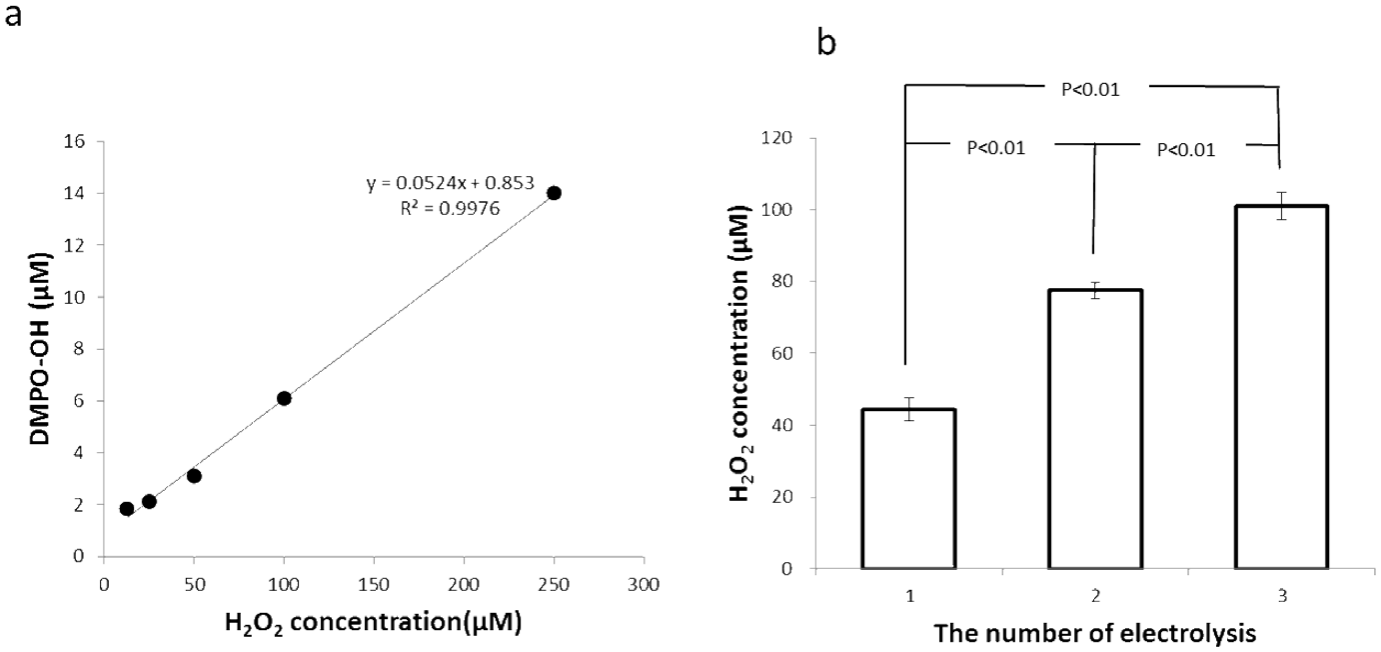
Determination of hydrogen peroxide concentrations. Calibration curve for determining hydrogen peroxide concentration (a) and hydrogen peroxide concentrations in AEW prepared by single and repeated electrolyses of 1% NaCl solution (b). Each value in the graph in (b) represents the mean ± standard deviation (*n* = 3). Tukey–Kramer's multiple comparison test was conducted for the hydrogen peroxide concentrations.

One of the putative origins of hydroxyl radicals is hydrogen peroxide. To determine the presence of hydrogen peroxide, ferrous sulfate (FeSO_4_), as a source of ferrous ions, was added to AEW to induce a Fenton reaction, as shown in the following equation:
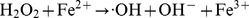
The ESR spectrum of AEW immediately after single electrolysis of 1% (w/v) NaCl solution and the spectrum after the addition of FeSO_4_ are shown in Fig. 3. The addition of FeSO_4_ (final concentration 45 µM) resulted in the appearance of a clear signal from DMPO-OH. To estimate the amount of hydroxyl radicals in the AEW, a calibration curve was prepared using given concentrations of hydrogen peroxide solutions, followed by the addition of FeSO_4_ and ESR determination of DMPO-OH (Fig. 4a). As shown in Fig. 4b, in which the concentrations of hydrogen peroxide in the AEWs are summarized, approximately 45 µM hydrogen peroxide was present in the singly electrolyzed AEW, and larger amounts of hydrogen peroxide were detected in the repeatedly electrolyzed AEWs (77 and 101 µM in the doubly and triply electrolyzed AEWs, respectively).

### Effects of hydroxyl radical scavengers and fetal bovine serum (FBS) on the bactericidal activity of AEW and free available chlorine concentration in AEW

**Figure 5:**
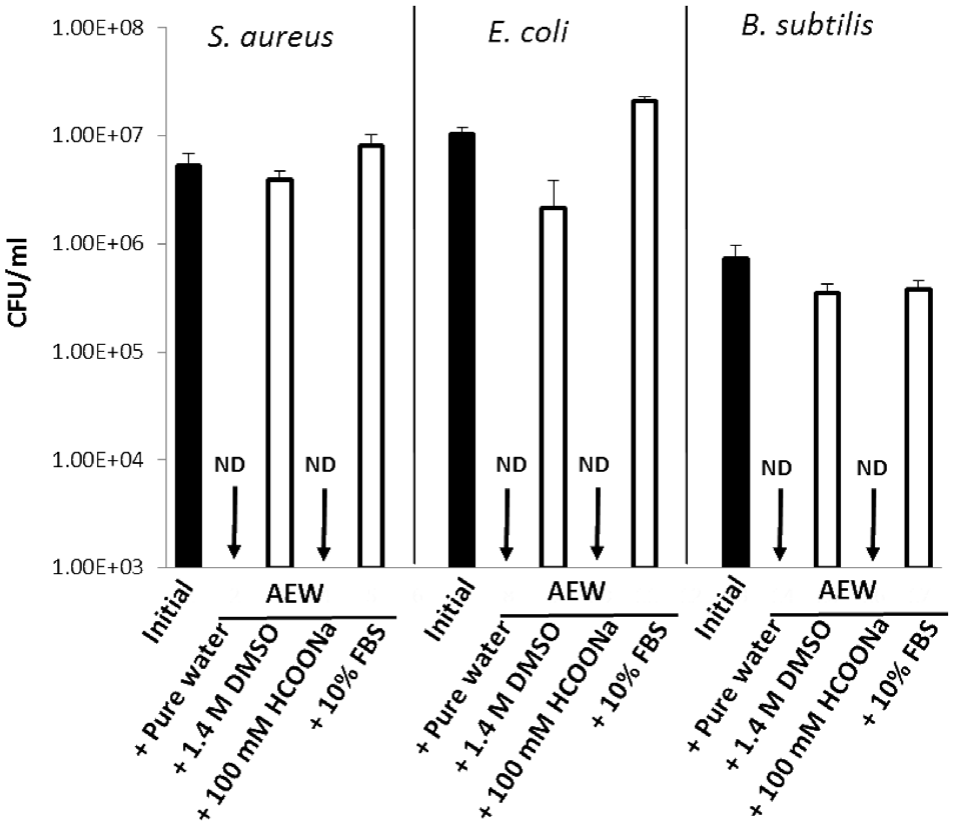
Effects of hydroxyl radical scavengers and fetal bovine serum on AEW bactericidal activity and free available chlorine concentration. DMSO and HCOONa were used as hydroxyl radical scavengers. FBS stands for fetal bovine serum. The AEW was prepared by single electrolysis of 0.1% (w/v) NaCl solution. Each value represents the mean+standard deviation (*n* = 2 or 3).

**Figure 6:**
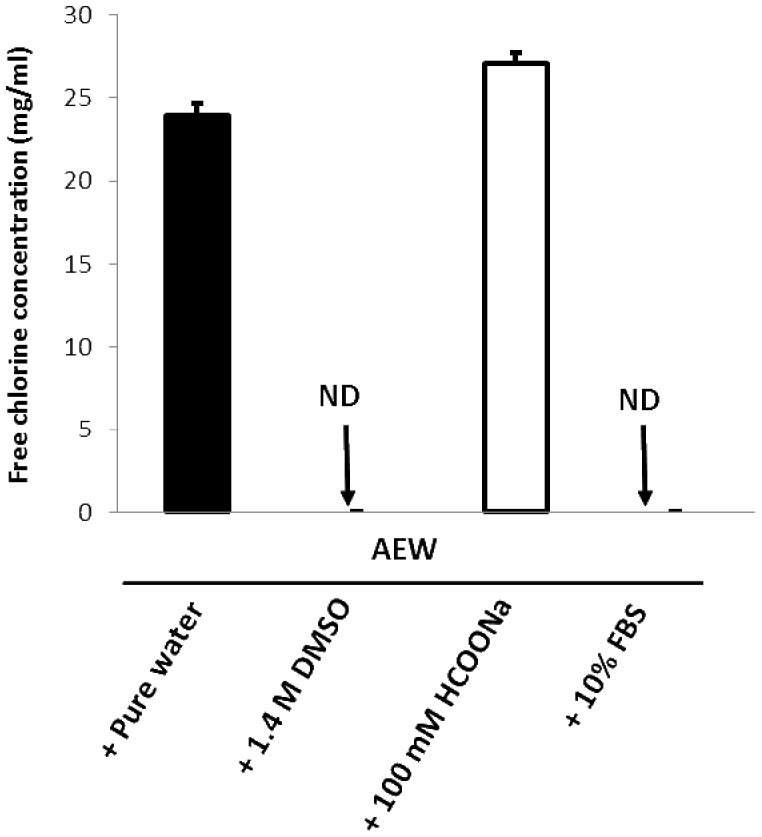
Effects of hydroxyl radical scavengers on free available chlorine concentration in AEW. DMSO and HCOONa were used as hydroxyl radical scavengers. FBS stands for fetal bovine serum. The AEW was prepared by single electrolysis of 0.1% (w/v) NaCl solution. Each value represents the mean+standard deviation (*n* = 2 or 3).

**Figure 7:**
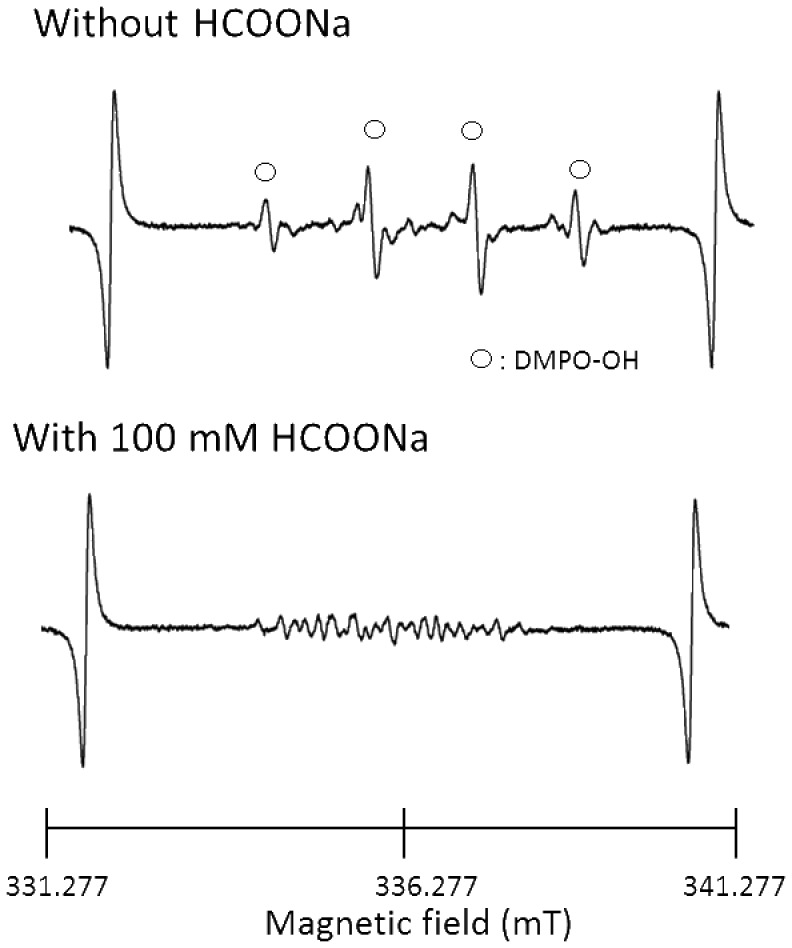
ESR spectra of AEW prepared by triple electrolyses of 1% NaCl solution. Spectra in the absence and presence of HCOONa are shown.

To examine whether the major contributor to the bactericidal activity of AEW is the hydroxyl radical, hydroxyl radical scavengers were added to AEW, followed by bactericidal assay against *Staphylococcus aureus*, *Escherichia coli*, and *Bacillus subtilis*, a spore forming bacterial species (Fig. 5). AEW prepared by single electrolysis of 0.1% (w/v) NaCl solution killed *S. aureus* and *E. coli* within 5–10 s, and *B. subtilis* within 10 min with at least three logarithmic reductions of viable bacterial cells. Although the addition of 100 mM sodium formate (HCOONa) did not destroy the bactericidal activity of the AEW, the addition of 1.4 M dimethyl sulfoxide (DMSO) destroyed the activity. The addition of FBS (10%, w/v of final concentration) completely destroyed the activity of the AEW. If HCOONa has antibacterial potential rather than hydroxyl radical scavenging activity, similar result would be obtained. Thus, antibacterial effect of HCOONa was examined. As a result, 100 mM HCOONa showed no bactericidal activity against the three bacterial species (data not shown). To examine whether the hydroxyl radical scavengers and FBS interfere with hypochlorous acid, the free available chlorine concentration was determined (Fig. 6). A close relationship was observed between the effects on the bactericidal activity and on the free available chlorine concentration; 100 mM HCOONa did not affect the concentration of free available chlorine, whereas both 1.4 M DMSO and 10% (w/v) FBS reduced the concentration to a non-detectable level (<0.01 mg/L). ESR spectra showing the effects of 100 mM HCOONa on the DMPO-OH signal observed in AEW obtained by the triple electrolyses of 1% (w/v) NaCl solution are shown in Fig. 7. The addition of 100 mM HCOONa reduced the signal to a non-detectable level.

## Discussion

Since the pH values decreased and both the ORP values and residual chlorine concentrations increased on repeated electrolysis (Tables 1 and 2), and, in particular, the residual chlorine concentration significantly increased as a result of increasing the concentration of NaCl, it is suggested that the increase in the amount of hypochlorous acid in the AEW depended not only on the number of electrolyses but also on the concentration of NaCl. The ESR analyses of the AEWs shown in Tables 1 and 2 reveal that the intensity of the DMPO-OH signal was clearly augmented each time the electrolysis of 1% NaCl solution was repeated (Fig. 1). Furthermore, despite the extremely short half-life of the hydroxyl radical (less than 10^−7^ s in a liquid) [29]–[31], DMPO-OH was detected even after storage for 1 h under light-shielding conditions (Fig. 2), suggesting that hydroxyl radicals were continuously formed in the AEW after electrolysis. This finding seems to support the idea that hydrogen peroxide exists in AEW as a source of hydroxyl radicals. To confirm this idea, FeSO_4_ was added to AEW to induce a Fenton reaction. As shown in Fig. 3, the addition of FeSO_4_ resulted in augmentation of the DMPO-OH signal intensity in AEW, indicating that the amount of hydroxyl radicals increased as a result of a Fenton reaction between hydrogen peroxide and ferrous ions. To estimate the amount of hydrogen peroxide in the AEW, a calibration curve was prepared using different concentrations of hydrogen peroxide solutions, followed by induction of a Fenton reaction (Fig. 4a). As a result, the amount of hydrogen peroxide increased with each electrolysis (Fig. 4b), which was consistent with the amount of hydroxyl radicals, as indicated by the increase in the DMPO-OH signal intensity when 1% (w/v) NaCl solution was repeatedly electrolyzed (Fig. 1). These results support the idea that hydrogen peroxide is a source of hydroxyl radials in AEW. Based on the results for AEW obtained by triple electrolyses of 1% (w/v) NaCl solution (Figs. 1 and 4b), a hydroxyl radical concentration of around 6 µM was estimated to be generated from 100 µM hydrogen peroxide. Since it was confirmed that dissolved oxygen was present in AEW at around 0.25 to 0.35 mM level which would be substantial for the formation of around a dozen to 100 µM hydrogen peroxide, hydrogen peroxide would be originated from dissolved oxygen. Further study is needed to clarify the involvement of dissolved oxygen in the hydrogen peroxide formation.

Regarding the active agents in AEW, it was confirmed that hypochlorous acid, but not the hydroxyl radical, is a major contributor to the bactericidal activity of AEW. Both DMSO and HCOONa can scavenge hydroxyl radicals [32]–[35], but the former canceled the activity of AEW, whereas the latter did not (Fig. 5). Since HCOONa did not exert bactericidal effect, direct interaction of HCOONa with the bacterial species tested would be negligible. This conflicting result is probably attributable to the interaction of DMSO and hypochlorous acid, since the addition of DMSO, but not of HCOONa, resulted in the complete depletion of free available chlorine in AEW (Fig. 6). Similarly to DMSO, FBS canceled the activity of AEW (Fig. 5), and this was also probably attributable to the depletion of free available chlorine, as reported in previous studies [36], [37] in which the bactericidal activity of AEW was shown to be correlated with the concentration of hypochlorous acid, and the activity was shown to be attenuated in the presence of organic materials such as proteins and amino acids through quick transformation of free available chlorine into N-chloro compounds.

The present study clearly revealed the presence of hydrogen peroxide in AEW, which in turn results in hydroxyl radical formation. However, although hydroxyl radicals can exert potent bactericidal activity, as shown in our previous studies [38], [39], where hydroxyl radicals generated by photolysis of hydrogen peroxide exerted potent bactericidal activity against various pathogenic bacterial species, the major contributor in AEW is hypochlorous acid, not the hydroxyl radicals, since HCOONa, a hydroxyl radical scavenger, failed to cancel the bactericidal activity of AEW, even though it reduced the DMPO-OH signal to a non-detectable level (Fig. 7). In contrast, DMSO and FBS canceled the bactericidal activity of AEW, accompanied by the complete depletion of free available chlorine, suggesting that degradation of hypochlorous acid caused by both FBS and DMSO destroyed the bactericidal activity.

## Materials and Methods

### Reagents

Reagents were purchased from the following sources: DMPO from Labotec (Tokyo, Japan), hydrogen peroxide from Santoku Chemical Industries (Tokyo, Japan), TEMPOL from Sigma Aldrich (St. Louis, MO, USA), NaCl, FeSO_4_, DMSO, and HCOONa from Wako Pure Chemical Industries (Osaka, Japan). All the reagents used were of analytical grade.

### Preparation of acid electrolyzed water (AEW)

Two different concentrations of NaCl solutions [0.1 and 1% (w/v)] were electrolyzed for 15 min using a batch-type electrolyzed water generator (Mini Super Water JED-007, Altec Janix, Kanagawa, Japan) at a regular AC voltage of 100 V and a rated current of 0.6 A. The characteristic values of the resultant AEWs were determined using a pH/ORP meter (D-53, Horiba Ltd., Kyoto, Japan) for pH and ORP, and a residual chlorine meter (HI95771, Hanna Instruments JAPAN, Tokyo, Japan) for residual chlorine (combined available chlorine+free available chlorine) concentrations. In some cases, the resultant AEW was subjected to one or two further electrolysis procedures. Since dissolved oxygen could be a key for the source of hydrogen peroxide, quantitative analysis of dissolved oxygen in AEW was also conducted using an oxygen sensor (Microx TX3, PreSens, Regensberg, Germany) in a separate experiment. The AEW stability was also examined after storage for 1 h at room temperature under light-shielding conditions.

### Determination of hydroxyl radicals by ESR spin-trapping technique

An aliquot (180 µL) of AEW was mixed with 20 µL of 8.9 M DMPO for 10 s. Immediately after mixing, the mixture was transferred to an ESR spectrometry cell, and the ESR measurements were started after 30 s on an X-band ESR spectrometer (JES-FA-100; JEOL, Tokyo, Japan). Regarding the concentration of DMPO, since the half-life of hydroxyl radical is extremely short as 10^−7^ s or less [29], [31], sufficient concentration of DMPO is necessary to trap all the hydroxyl radical generated. Our previous study revealed that 300 mM or more DMPO is necessary to trap several dozen µM hydroxyl radical [40], so that DMPO at the final concentration of 890 mM was used in the present study. TEMPOL (20 µM) was used as a standard sample to calculate the concentration of DMPO-OH, a spin adduct of DMPO and a hydroxyl radical. The measurement conditions were as follows: field sweep, 330.50 to 340.50 mT; field modulation frequency, 100 kHz; field modulation width, 0.1 mT; amplitude, 200; sweep time, 2 min; time constant, 0.03 s; microwave frequency, 9.420 GHz; microwave power, 4 mW. All experiments were performed in triplicate at room temperature.

### Induction of Fenton reaction as a tool for determination of hydrogen peroxide

To determine the hydrogen peroxide in AEW, ferrous iron was added to the AEW to induce a Fenton reaction. An aliquot (180 µL) of AEW was mixed with 20 µL of 8.9 M DMPO and 20 µL of 0.5 mM FeSO_4_ for 10 s. Immediately after mixing, the mixture was transferred to an ESR spectrometry cell, and the ESR measurements were started after 30 s on an X-band ESR spectrometer. To quantitatively analyze the hydrogen peroxide in AEW, hydrogen peroxide solutions of given concentrations were prepared, and 180 µL of each hydrogen peroxide solution were mixed with 20 µL of 8.9 M DMPO and 20 µL of 0.5 mM FeSO_4_ for 10 s, followed by ESR analysis as described above, to obtain a calibration curve for hydrogen peroxide. For statistical analysis of the hydrogen peroxide concentrations, Tukey–Kramer's multiple comparison test for pairwise comparisons was conducted. *P* values of <0.05 were considered significant.

### Effects of hydroxyl radical scavengers and fetal bovine serum (FBS) on bactericidal activity of AEW and free available chlorine concentration in AEW

Stock culture strains of *S. aureus* JCM 2413, *E. coli* JCM 5491, and *B. subtilis* JCM 1465 were purchased from Japan Collection of Microorganisms, RIKEN BioResource Center (Wako, Japan). *S. aureus* and *E. coli* were cultured on brain–heart infusion (BHI) agar (Becton, Dickinson Company, Sparks, MD, USA) at 37°C overnight. *B. subtilis*, a spore forming bacterial species, cultured on BHI agar at 37°C for 1 week was harvested and heated at 65°C for 30 min, and then stored at 4°C until assayed. Then the bacterial cells were suspended in sterile physiological saline at 1.0–3.0×10^8^ cells/mL as an inoculum. Ten microliters of each inoculum were mixed with 1 mL of AEW for 5 to 10 s against *S. aureus* and *E. coli*, and for 10 min against *B. subtilis*. Then the mixture was diluted 10 times with BHI broth, and 200 µL of the diluted mixture were further incubated on a BHI agar plate at 37°C for 2 d for determining the number of viable bacterial cells. To confirm whether the bactericidal activity of AEW is in any way attributable to hydroxyl radicals, the effects of DMSO and HCOONa, hydroxyl radical scavengers, were examined. One hundred microliters of 14 M DMSO or 1 M HCOONa were added to 890 µL of AEW, followed by addition of 10 µL of the bacterial suspension. Then the bactericidal tests were similarly performed. In addition, to confirm whether the bactericidal activity of AEW depends on hypochlorous acid, the bactericidal activity of AEW was similarly examined in the presence of 10% (w/v) FBS (Life Technologies Corp., Carlsbad, CA, USA) since the bactericidal activity of AEW could be attenuated by organic materials such as proteins and amino acids through rapid transformation of free available chlorine into N-chloro compounds [35]. To confirm that HCOONa does not possess antibacterial potential, 100 mM HCOONa instead of AEW was similarly subjected to the bactericidal tests. Furthermore, to examine the relationship between the bactericidal activity and degradation of hypochlorous acid, the concentrations of free available chlorine, including Cl_2_, HClO, and ClO^−^, in AEW with 1.4 M DMSO, 100 mM HCOONa, or 10% (w/v) FBS were determined by the *N*,*N*′-diethyl-*p*-phenylenediamine (DPD) standard method [37], [41].

## References

[1] Northcutt J, Smith D, Ingram KD, Hinton A Jr, Musgrove M (2007) Recovery of bacteria from broiler carcasses after spray washing with acidified electrolyzed water or sodium hypochlorite solutions. Poult Sci 86: 2239–2244. 17878456 10.1093/ps/86.10.2239

[2] Fabrizio KA, Cutter CN (2004) Comparison of electrolyzed oxidizing water with other antimicrobial interventions to reduce pathogens on fresh pork. Meat Sci 68: 463–468. 22062415 10.1016/j.meatsci.2004.04.013

[3] Guentzel JL, Liang Lam K, Callan MA, Emmons SA, Dunham VL (2008) Reduction of bacteria on spinach, lettuce, and surfaces in food service areas using neutral electrolyzed oxidizing water. Food Microbiol 25: 36–41. 17993375 10.1016/j.fm.2007.08.003

[4] Cao W, Zhu ZW, Shi ZX, Wang CY, Li BM (2009) Efficiency of slightly acidic electrolyzed water for inactivation of Salmonella enteritidis and its contaminated shell eggs. Int J Food Microbiol 130: 88–93. 19185376 10.1016/j.ijfoodmicro.2008.12.021

[5] Park YB, Guo JY, Rahman SM, Ahn J, Oh DH (2009) Synergistic effect of electrolyzed water and citric Acid against bacillus cereus cells and spores on cereal grains. J Food Sci 74: M185–189. 19490337 10.1111/j.1750-3841.2009.01139.x

[6] Lu ZH, Zhang Y, Li LT, Curtis RB, Kong XL, et al (2010) Inhibition of microbial growth and enrichment of gamma-aminobutyric acid during germination of brown rice by electrolyzed oxidizing water. J Food Prot 73: 483–487. 20202333 10.4315/0362-028x-73.3.483

[7] Rahman SM, Ding T, Oh DH (2010) Effectiveness of low concentration electrolyzed water to inactivate foodborne pathogens under different environmental conditions. Int J Food Microbiol 139: 147–153. 20385418 10.1016/j.ijfoodmicro.2010.03.020

[8] Rahman SM, Jin YG, Oh DH (2011) Combination treatment of alkaline electrolyzed water and citric acid with mild heat to ensure microbial safety, shelf-life and sensory quality of shredded carrots. Food Microbiol 28: 484–491. 21356455 10.1016/j.fm.2010.10.006

[9] Lee JH, Rhee PL, Kim JH, Kim JJ, Paik SW, et al (2004) Efficacy of electrolyzed acid water in reprocessing patient-used flexible upper endoscopes: Comparison with 2% alkaline glutaraldehyde. J Gastroenterol Hepatol 19: 897–903. 15242493 10.1111/j.1440-1746.2004.03375.x

[10] Tanaka N, Fujisawa T, Daimon T, Fujiwara K, Yamamoto M, et al (2000) The use of electrolyzed solutions for the cleaning and disinfecting of dialyzers. Artif Organs 24: 921–928. 11121970 10.1046/j.1525-1594.2000.06611.x

[11] Nagamatsu Y, Tajima K, Kakigawa H, Kozono Y (2001) Application of electrolyzed acid water to sterilization of denture base part 1. Examination of sterilization effects on resin plate. Dent Mater J 20: 148–155. 11523978 10.4012/dmj.20.148

[12] Kiura H, Sano K, Morimatsu S, Nakano T, Morita C, et al (2002) Bactericidal activity of electrolyzed acid water from solution containing sodium chloride at low concentration, in comparison with that at high concentration. J Microbiol Methods 49: 285–293. 11869793 10.1016/s0167-7012(01)00385-2

[13] Sharma RR, Demirci A (2003) Treatment of *Escherichia coli* O157:H7 inoculated alfalfa seeds and sprouts with electrolyzed oxidizing water. Int J Food Microbiol 86: 231–237. 12915034 10.1016/s0168-1605(02)00549-4

[14] Nisola GM, Yang X, Cho E, Han M, Lee C, et al (2011) Disinfection performances of stored acidic and neutral electrolyzed waters generated from brine solution. J Environ Sci Health A Tox Hazard Subst Environ Eng 46: 263–270. 21308597 10.1080/10934529.2011.535428

[15] Rodriguez-Garcia O, Gonzalez-Romero VM, Fernandez-Escartin E (2011) Reduction of *Salmonella enterica*, *Escherichia coli* O157:H7, and *Listeria monocytogenes* with electrolyzed oxidizing water on inoculated hass avocados (*Persea americana var. Hass*). J Food Prot 74: 1552–1557. 21902927 10.4315/0362-028X.JFP-11-047

[16] Feliciano L, Lee J, Pascall MA (2012) Transmission electron microscopic analysis showing structural changes to bacterial cells treated with electrolyzed water and an acidic sanitizer. J Food Sci 77: M182–187. 22515246 10.1111/j.1750-3841.2012.02633.x

[17] Handojo A, Lee J, Hipp J, Pascall MA (2009) Efficacy of electrolyzed water and an acidic formulation compared with regularly used chemical sanitizers for tableware sanitization during mechanical and manual ware-washing protocols. J Food Prot 72: 1315–1320. 19610348 10.4315/0362-028x-72.6.1315

[18] Becking LGMB, Kaplan IR, Moore D (1960) Limits of the natural environment in terms of pH and oxidation-reduction potentials. J Geolog 68: 243–284.

[19] Hotta K (2000) Acid electrolyzed saline solution: Its function and medical application. J Jpn Soc Intensive Care Med 7: 97–105 (in Japanese).

[20] Al-Haq MI, Sugiyama J, Isobe S (2005) Applications of electrolyzed water in qgriculture & food Industries. Food Sci Technol Res 11: 135–150.

[21] Albrich JM, Gilbaugh JH 3rd, Callahan KB, Hurst JK (1986) Effects of the putative neutrophil-generated toxin, hypochlorous acid, on membrane permeability and transport systems of Escherichia coli. J Clin Invest 78: 177–184. 3013936 10.1172/JCI112548 PMC329547

[22] Barrette WC Jr, Hannum DM, Wheeler WD, Hurst JK (1989) General mechanism for the bacterial toxicity of hypochlorous acid: abolition of ATP production. Biochemistry 28: 9172–9178. 2557918 10.1021/bi00449a032

[23] Hurst JK, Barrette WC Jr, Michel BR, Rosen H (1991) Hypochlorous acid and myeloperoxidase-catalyzed oxidation of iron-sulfur clusters in bacterial respiratory dehydrogenases. Eur J Biochem 202: 1275–1282. 1662610 10.1111/j.1432-1033.1991.tb16500.x

[24] Yonemori S, Takimoto Y, Min KH, Jitsugiri Y, Simohira T, et al (1997) Analysis of hydroxyl radical generated in electrolyzed strong acid aqueous solution by electron spin resonance spectroscopy. Nippon Kagaku Kaishi 7: 497–501 (in Japanese).

[25] Stan SD, Daeschel MA (2005) 5,5-Dimethyl-2-pyrrolidone-N-oxyl formation in electron spin resonance studies of electrolyzed NaCl solution using 5,5-dimethyl-1-pyrroline-N-oxide as a spin trapping agent. J Agric Food Chem 53: 4906–4910. 15941334 10.1021/jf047918k

[26] Tsuchiya K, Hotta K (2004) Characterization of chemical factors as the bactericidal basis of acidic Denkaisui. J Functional Water 2: 75–80 (in Japanese).

[27] Arakawa H, Maeda M, Okubo S, Shimamura T (2004) Role of hydrogen peroxide in bactericidal action of catechin. Biol Pharm Bull 27: 277–281. 14993788 10.1248/bpb.27.277

[28] Buettner GR (1987) Spin trapping: ESR parameters of spin adducts. Free Radic Biol Med 3: 259–303. 2826304 10.1016/s0891-5849(87)80033-3

[29] Pryor WA (1986) Oxy-radicals and related species: their formation, lifetimes, and reactions. Annu Rev Physiol 48: 657–667. 3010829 10.1146/annurev.ph.48.030186.003301

[30] Sies H, Stahl W, Sundquist AR (1992) Antioxidant functions of vitamins. Vitamins E and C, beta-carotene, and other carotenoids. Ann N Y Acad Sci 669: 7–20. 1444060 10.1111/j.1749-6632.1992.tb17085.x

[31] Redmond RW, Kochevar IE (2006) Spatially resolved cellular responses to singlet oxygen. Photochem Photobiol 82: 1178–1186. 16740059 10.1562/2006-04-14-IR-874

[32] Panganamala RV, Sharma HM, Heikkila RE, Geer JC, Cornwell DG (1976) Role of hydroxyl radical scavengers dimethyl sulfoxide, alcohols and methional in the inhibition of prostaglandin biosynthesis. Prostaglandins 11: 599–607. 822477 10.1016/0090-6980(76)90063-0

[33] Suthanthiran M, Solomon SD, Williams PS, Rubin AL, Novogrodsky A, et al (1984) Hydroxyl radical scavengers inhibit human natural killer cell activity. Nature 307: 276–278. 6694728 10.1038/307276a0

[34] Winterbourn CC (1987) The ability of scavengers to distinguish OH^.^ production in the iron-catalyzed Haber-Weiss reaction: comparison of four assays for OH^.^ . Free Radic Biol Med 3: 33–39. 3040537 10.1016/0891-5849(87)90037-2

[35] Herskind C, Westergaard O (1988) Variable protection by OH scavengers against radiation-induced inactivation of isolated transcriptionally active chromatin: the influence of secondary radicals. Radiat Res 1114: 28–41. 2832871

[36] Nakagawara S, Goto T, Nara M, Ozawa Y, Hotta K, et al (1998) Spectroscopic characterization and the pH dependence of bactericidal activity of the aqueous chlorine solution. Anal Sci 14: 691–698.

[37] Oomori T, Oka T, Inuta T, Arata Y (2000) The efficiency of disinfection of acidic electrolyzed water in the presence of organic materials. Anal Sci 16: 365–369.

[38] Ikai H, Nakamura K, Shirato M, Kanno T, Iwasawa A, et al (2010) Photolysis of hydrogen peroxide, an effective disinfection system via hydroxyl radical formation. Antimicrob Agents Chemother 54: 5086–5091. 20921319 10.1128/AAC.00751-10 PMC2981275

[39] Shirato M, Ikai H, Nakamura K, Hayashi E, Kanno T, et al (2012) Synergistic effect of thermal energy on bactericidal action of photolysis of H_2_O_2_ in relation to acceleration of hydroxyl radical generation. Antimicrob Agents Chemother 56: 295–301. 22024818 10.1128/AAC.05158-11 PMC3256047

[40] Nakamura K, Kanno T, Ikai H, Sato E, Mokudai T, et al (2010) Reevaluation of quantitative ESR spin trapping analysis of hydroxyl radical by applying sonolysis of water as a model system. Bull Chem Soc Jpn 83: 1037–1046.

[41] Standard Methods for the Examination of water and Wastewater, 16th ed., APHA, AWWA, WPCF, Washington. 1985, pp. 309–314.

